# SciGeneX: enhancing transcriptional analysis through gene module detection in single-cell and spatial transcriptomics data

**DOI:** 10.1093/nargab/lqaf043

**Published:** 2025-04-17

**Authors:** Julie Bavais, Jessica Chevallier, Lionel Spinelli, Serge A van de Pavert, Denis Puthier

**Affiliations:** Aix-Marseille Univ, INSERM, TAGC, Turing Centre for Living systems, 13288 Marseille, France; Aix-Marseille Univ, CNRS, INSERM, CIML, Turing Centre for Living systems, 13009 Marseille, France; Aix-Marseille Univ, INSERM, TAGC, Turing Centre for Living systems, 13288 Marseille, France; Aix-Marseille Univ, CNRS, INSERM, CIML, Turing Centre for Living systems, 13009 Marseille, France; Aix-Marseille Univ, INSERM, TAGC, Turing Centre for Living systems, 13288 Marseille, France; Aix-Marseille Univ, CNRS, INSERM, CIML, Turing Centre for Living systems, 13009 Marseille, France; Aix-Marseille Univ, CNRS, INSERM, CIML, Turing Centre for Living systems, 13009 Marseille, France; Aix-Marseille Univ, INSERM, TAGC, MarMaRa Institute, Turing Centre for Living systems, Transcriptomics and Genomics Marseille Luminy (TGML), 13288 Marseille, France

## Abstract

The standard pipeline to analyze single-cell RNA-seq or spatial transcriptomics data focuses on a gene-centric approach that overlooks the collective behavior of genes. However, understanding cell populations necessitates recognizing intricate combinations of activated and repressed pathways. Therefore, a broader view of gene behavior offers more accurate insights into cellular heterogeneity in single-cell or spatial transcriptomics data. Here, we describe SciGeneX (Single-cell informative Gene eXplorer), a R package implementing a neighborhood analysis and a graph partitioning method to generate co-expression gene modules. These modules, whether shared or restricted to cell populations, collectively reflect cellular heterogeneity. Their combinations are able to highlight specific cell populations, even rare ones. SciGeneX uncovers rare and novel cell populations that were not observed before in human thymus spatial transcriptomics data. We show that SciGeneX outperforms existing methods on both artificial and experimental datasets. Overall, SciGeneX will aid in unravelling cellular and molecular diversity in single-cell and spatial transcriptomics studies.

## Introduction

Advancements in next-generation sequencing technologies have revolutionized the transcriptomics field, offering detailed information on individual cells [1]. Among these technologies, single-cell RNA-seq (scRNA-seq) and spatial transcriptomics (STs) have emerged as groundbreaking technologies for investigating cellular heterogeneity within complex tissues [2, 3]. ST is anticipated to revolutionize biology by providing insights into the intricate interplay of genes and cells within their native microenvironments [4, 5]. Numerous tools have been developed to analyze scRNA-seq [6] or ST [7, 8] with Seurat [9] and Scanpy [10] as the predominant choice in current studies [11] and have become a standard library for scRNA-seq and ST data analysis [12]. The standard pipeline to analyze scRNA-seq or ST data starts with pre-processing steps to remove non-biological sources of variations with quality control [13] and cell counts normalization [14]. Feature selection extracts informative genes [15], which are then reduced to a latent space using principal component analysis (PCA), and unsupervised cell clustering is performed in the latent space [16]. Differential gene expression analysis identifies genes specific to clusters [17], enabling assignment of distinct cell types or states. Cells are subsequently visualized in two-dimensional space using t-distributed stochastic neighbor embedding or uniform manifold approximation and projection (UMAP) [18].

Identifying the optimal cell partitioning remains a challenge that requires multiple empirical clustering attempts, along with the identification of differentially expressed genes (DEGs). Moreover, DEG quality is intricately linked to the cell clustering quality and failure to identify cell populations such as rare ones prevents DEGs identification for this population [19]. Additionally, the dual use of gene information for both partitioning the cells or spots and identifying DEGs leads to double-dipping, inducing a high rate of false positive [20]. Finally, this standard approach firstly focuses on analyzing genes on an individual basis to dissect cellular or spot intricacies. However, this gene-centric analysis, while informative, falls short of encapsulating the whole comprehensive complexity inherent in cellular biology. The standard pipeline, by focusing on a gene-by-gene study, overlooks the collective behavior of genes and may inadvertently neglect those that, while less variable individually, could collectively define critical cellular states.

Knowing that cell populations emerge from complex activation and repression of gene expression, cell populations could be considered as combinations of these regulatory pathways providing a more comprehensive perspective. A promising alternative to the standard pipeline is to first cluster genes into co-expression modules and then group the cells according to their modules usage [21]. Indeed, in contrast to gene clustering, which can be evaluated with functional enrichment, the cell or spot clustering approach used in the classical pipeline does not rely on any biological indicator. It is within this biological context that we developed SciGeneX (Single-cell informative Gene eXplorer), a R package implementing an algorithm to generate co-expression gene modules. Unlike the standard pipeline, SciGeneX does not limit itself to a gene-centric analysis but instead delves into the broader panorama of gene co-expression. SciGeneX opens up new possibilities for understanding cellular dynamics and heterogeneity, reflecting a deeper commitment to capturing the intricate relationships between genes. This ultimately leading to more meaningful insights into the biological underpinnings of scRNA-seq and ST data.

The SciGeneX algorithm is an adaptation of the DBF-MCL algorithm, which we previously developed in the context of microarray analysis [22]. SciGeneX implements a neighborhood analysis to eliminate non-co-expressed genes across cell populations and partition the remaining genes into gene modules using the Markov Cluster Algorithm (MCL) [23]. We benchmarked the SciGeneX neighborhood analysis against classical feature selection methods using a set of artificial and real datasets and showed that overall SciGeneX outperformed the latter. Our analysis demonstrated that the gene modules generated by SciGeneX are highly resolutive and enriched in specific biological processes using a small [24] and a large dataset [25] containing both mature and differentiating cells. These modules, reminiscent of specific cell markers, cell states, and numerous signaling pathways, can be combined to reveal biologically relevant cell groups independently of the classical scRNA-seq analysis pipeline. SciGeneX shows its ability to reveal previously undetected rare cell populations and associated marker genes. Finally, we demonstrate that the SciGeneX approach translates to Visium ST data and can elucidate biologically meaningful topological molecular profiles. To store the co-expression gene modules, we have implemented an S4 R class named “ClusterSet”. This implementation empowers the users to access various functionalities to visualize, characterize, and manipulate the discovered gene modules.

Overall, our results indicate that SciGeneX introduces a promising approach to scRNA-seq and ST data analysis. Through its innovative co-expression module generation, SciGeneX outperforms conventional methods, demonstrating superior efficacy in identifying and extracting informative genes. SciGeneX provides highly resolved gene modules, enriched in specific biological processes, capable of revealing previously undetected rare cell populations and associated markers in both scRNA-seq and ST data.

## Materials and methods

### The SciGeneX algorithm

**Figure 1.**
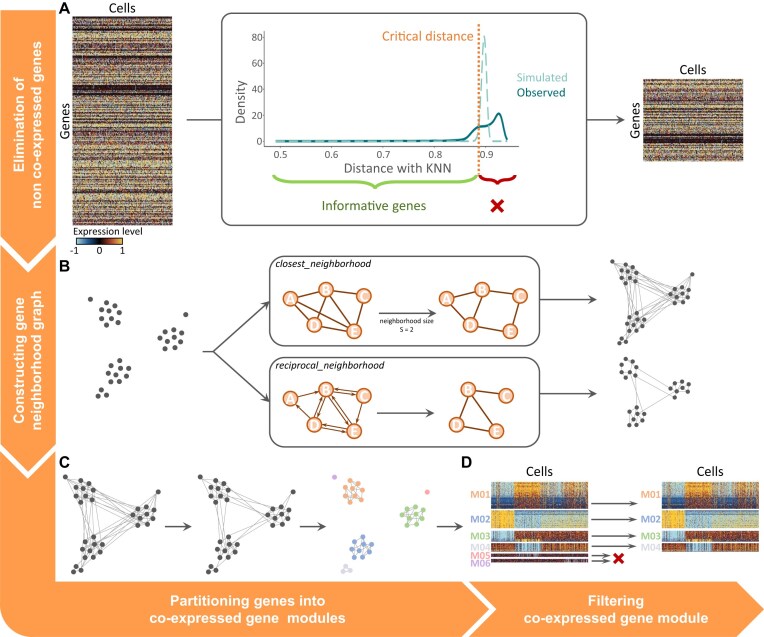
Overview of SciGeneX algorithm. The SciGeneX algorithm consists of four consecutive steps. (**A**) The algorithm first eliminates non co-expressed genes employing a density-based filtering approach. It involves comparing the gene–gene distance matrix, typically using the Pearson correlation coefficient, against a null hypothesis of random gene distribution. Distances with the K nearest neighbor (DKNN) are computed, and a threshold is established to eliminate genes that do not co-express with other genes. (**B**) Then, the SciGeneX algorithm constructs a gene’s neighborhood graph. Two methods are available. The closest_neighborhood method establishes edges based on the size (**S**) of each gene’s closest neighborhood while the reciprocal_method considers reciprocal relationships within a neighborhood of size K. (**C**) Gene selected are then clustered into co-expression modules using the MCL algorithm. (**D**) Finally, co-expression modules are filtered typically based on module size or standard deviation.

The SciGeneX algorithm aims to identify and select modules of co-expressed genes. To do so, instead of examining cells in gene dimensions as in the conventional approach, SciGeneX analyzes genes in cell dimensions. In this space, genes whose expression is similar across cells are assumed to be close to each other. We illustrate genes in a 2D space to facilitate this visualization in Fig. 1. From a more global point of view, sets of highly co-expressed genes corresponding to biological programs activated differentially between cells can generate dense regions of genes.

The SciGeneX algorithm aims to detect those groups of highly co-expressed biologically meaningful genes. It is divided into four main steps: (i) elimination of genes that do not co-express with other genes, (ii) construction of a gene’s neighborhood graph, (iii) identification of modules of co-expressed genes, and (iv) filtering of modules of co-expressed genes.

The algorithm initially examines the distances between genes in a cell dimension, eliminating genes that are distant to each other, considering them as non-co-expressed across cells. To do so, the algorithm compares the distance of the nearest neighbors to the distribution of similar distances computed under the null hypothesis of random distribution of genes across the cell space. The genes having a distance too similar to the random distribution are removed from the downstream analysis. More precisely, the algorithm starts by computing a gene–gene distance matrix based, by default, on the Pearson correlation coefficient. This matrix reflects the relationships and similarities between different genes in terms of expression patterns. Alternative distance metrics are proposed in the package like Euclidean distances, Spearman correlation coefficient, and cosine distances. Using this matrix, the algorithm computes the DKNN for each gene. This step quantifies the proximity of a gene to its closest neighbors in the gene expression space. The distribution of these distances for all genes is most often a long-tail distribution (Fig. 1), suggesting the presence of co-expressed genes within the dataset. Next, to determine a threshold on DKNN above which genes will not be considered as co-expressing with other genes, SciGeneX computes the distribution of the DKNN distances under the hypothesis of no co-regulated genes. The algorithm uses random distance matrix permutations without replacement to compute null-hypothesis distance distribution and compares the observed distances to the simulated ones. This comparison aims to estimate the false discovery rate (FDR), which helps control the rate of false positives in the subsequent identification of co-expressed genes. From the simulated distances, SciGeneX determines a DKNN threshold below which distances are considered indicative of the presence of co-regulated genes. This threshold is determined based on the confidence level or the acceptable proportion of false positives. Genes with observed DKNN distances above this threshold are removed from the downstream analysis.

In the second step, the algorithm constructs a neighborhood graph where nodes represent genes. Edges in the graph can be defined using two methods. The first one, called closest_neighborhood, establishes an edge between genes A and B if B is part of the nearest neighborhood of size S for gene A (with S < K). The second one, called reciprocal_neighborhood, inspects the neighborhood of size K of all selected genes and puts an edge between two genes, A and B, if they are reciprocally in each other’s neighborhood. For this last method, we recommend using a higher K value, as more edges tend to be pruned using the reciprocity filter.

Finally, the algorithm searches to identify the densely connected regions of the neighborhood graph that may correspond to the highly-co-expressed genes across cells. To do so, the MCL algorithm is applied to the graph to create a partition of the genes and define gene modules. This algorithm simulates a random walk through the graph using expansion and inflation operators. This algorithm has successfully been used in various data analysis contexts including protein–protein interaction graph partitioning [26] or orthologous genes analysis [27, 28]. It is worth noting that MCL tends to produce several singleton clusters [29], thus, a filtering step, typically on module size or standard deviation of gene expression within a module, is subsequently applied.

The complete SciGeneX algorithm produces a list of gene modules, each module containing a set of genes that are highly co-expressed in cells and may correspond to biologically relevant processes that allow groups of cells to be distinguished by type or state.

### Implementation in R and analysis features

The SciGeneX algorithm has been implemented in a R package with a very easy-to-use API. The final co-expression gene modules are stored within an S4 object of the ClusterSet class, which has been implemented to offer functionalities for subsequent analyses. Users can utilize various features, such as (i) filtering options: filter based on criteria like gene number, gene names, or standard deviation; (ii) effortless module selection: easily select gene modules using the indexing function; (iii) visualization capabilities: visualize results through interactive and non-interactive heatmaps, where cells are arranged using a hierarchical clustering algorithm; (iv) Gene Ontology (GO) enrichment analysis: generate matrices for GO enrichment analysis across co-expression modules; (v) reference cell markers: create matrices of reference cell markers that correspond to gene modules; (vi) global expression visualization: visualize the global expression of a gene module on the cell UMAP; and (vii) export options: export results to Excel spreadsheet or an HTML report for convenient data sharing. All these functionalities provide users with a comprehensive set of tools for in-depth exploration and interpretation of their data, enhancing the analytical capabilities provided by SciGeneX.

### Methods evaluated for the benchmark

The DISP, variance-stabilizing transformation (VST), and sctransform methods all aim to select highly variable genes by identifying those with significant biological variability while accounting for technical noise. DISP (dispersion) compares the observed dispersion (variance normalized by the mean) of each gene to an expected mean-dispersion trend, selecting genes with the highest adjusted dispersion. VST stabilizes variance across expression levels by applying a regularized negative binomial model or a log transformation, then selects genes with the highest residual variance. Sctransform, on the other hand, uses a generalized linear model to explicitly model the relationship between variance and sequencing depth, selecting genes with high residual variance after adjusting for technical effects.

The singleCellHaystack method uses the Kullback-Leibler divergence to detect genes expressed in subsets of cells that are non-randomly distributed in a multidimensional space. For each gene, the method compares the spatial distribution of cells expressing the gene to a reference distribution of all cells. The Kullback-Leibler divergence is computed for each gene and compared to randomized data to assess statistical significance.

The M3Drop method selects genes by analyzing gene dropouts rate. It uses a Michaelis–Menten model to relate a gene mean expression to its dropout rate, calculating a key parameter for each gene. A *t*-test evaluates whether a gene dropout behavior significantly differs from the global trend.

The BigSur method is based on Pearson residuals, which measure the difference between observed and expected gene expression in each cell. The method calculates a modified version of these residuals by normalizing them for each cell’s UMI proportion and adjusting for stochasticity in gene expression. Genes with large squared residuals across cells are considered more variable and are selected.

CoGAPS and GeneNMF methods based their gene selection on non-negative matrix factorization. It decomposes the gene-cell expression matrix into two non-negative factor matrices, reducing complexity and extracting interpretable features. GeneNMF identifies meta-programs, which are robust gene programs consistently detected across multiple NMF runs and include genes in a meta-program if their cumulative weights contribute significantly to the total, determined by a threshold. CoGAPS employs a Bayesian approach using a Markov Chain Monte Carlo algorithm to generate gene modules.

### Artificial datasets

We used the SPARSim R package to generate a comprehensive set of 100 scRNA-seq artificial datasets of 1755 cells distributed across five clusters with 7204 genes. Input parameters, including gene expression level intensities, gene expression level variabilities, and sample library sizes, were estimated from the NK populations (*n* = 155) of the PBMC3k dataset using the SPARSim_estimate_parameter_from_data() function. Gene expression level intensities were defined as the mean of the normalized counts across samples by the SPARSim methodology while, gene expression level variabilities as the variance of normalized counts across samples. The library size was established as the UMI count per cell. Within each dataset, we simulated four sets of DEGs, encompassing 500, 300, 200, and 50 genes, with a fold change uniformly distributed between 4 and a maximum ranging from 10 to 100. The simulated parameters of DEGs (intensities, variabilities, and library size) were estimated using the SPARSim_create_DE_genes_parameter() function. Thus, a total of 10 datasets for each 10 fold-change increment were generated using the SPARSim_simulation() function. UMAP visualizations in Supplementary Fig. S2 were generated using the Seurat pipeline (version 4.3.0). The count matrix was first normalized using the NormalizeData() function. A total of 1050 variable genes were identified using the FindVariableFeatures() function, based on our simulation. We then performed PCA using the RunPCA() function to generate 50 principal components (PCs), which were subsequently used to create the UMAP visualization with the RunUMAP() function.

### Definition of true informative genes in Tabula Muris datasets

We downloaded the 12 droplet Seurat objects and their associated metadata from the Tabula Muris consortium website (https://tabula-muris.ds.czbiohub.org/) representing a range of tissues including bladder, heart and aorta, kidney, limb muscle, liver, lung, mammary gland, marrow, spleen, thymus, tongue, and trachea. Each Seurat object contains single-cell transcriptomic data with detailed annotations of cell types. We filtered out genes expressed in less than three cells for the subsequent analysis. Given the pre-defined cell type annotations, we identified for each tissue the cell population DEGs using four methods (Wilcoxon, *t*-test, bimod and MAST) from the FindAllMakers() function (Seurat version 4.3.0) using default parameter except for only.pos = TRUE and min.pct = 0.25. Each method provided us with a set of true informative genes, enabling us to evaluate performance without relying on a single test. Genes were then selected based on a minimal average log2 fold change of 0.5 and a maximum adjusted *p*-value of .05. These selected genes were considered the true informative genes for subsequent performance analysis.

### Evaluation of prediction performance

Artificial datasets. To predict the true informative genes in the artificial datasets, we applied both SciGeneX methods (closest_neighborhood and reciprocal_neighborhood) and eight existing methods (DISP, VST, sctransform, M3Drop, singleCellHaystack, BigSur, CoGAPS, and GeneNMF). We pre-processed the datasets to only retain genes expressed in at least four cells. To select genes using the SciGeneX methods, we employed the select_genes() function with the following parameters: distance_method=“pearson,” noise_level=0.05, and a FDR of 1e-4. The noise_level parameter defines the fraction of genes with high DKNN (i.e. noise) used to compute simulated DKNN values. A value of 0 means to use all genes whereas a value close to 1 means to use genes with high DKNN. For the closest_neighborhood method, we used *K* = 50, while we employed *K* = 100 for the reciprocal_neighborhood method. Genes were subsequently clustered into co-expression modules using the gene_clustering() function. Graphs were partitioned using an inflation of 1.2. Finally, co-expression modules with <10 genes were filtered out using the filter_cluster_size() function. Using the filter_nb_supporting_cells() only modules for which at least five cells expressed 20% of the gene from the module were selected. Coupled with this, a selection of 2000 genes was done with the DISP and VST methods using the FindVariableFeatures() function from the Seurat package (version 4.3.0) with default settings. For sctransform method, genes were selected using the SCTransform() function from Seurat package (version 4.3.0). We used the M3DropFeatureSelection() function to predict informative genes with M3Drop (version 1.20.0), the haystack() and show_result_haystack() functions for singleCellHaystack (verison 1.0.2) and the BigSur() function for BigSur gene prediction (version 1.0.1). For the CoGaps method, we set the parameters using the CogapsParams() and setDistributedParams() commands and generate the gene modules using the CoGAPS() command (version 3.5.6). Finally, for the GeneNMF method, we used the multiNMF() and getMetaPrograms() commands to obtain the gene modules (version 0.6.2). Subsequently, we computed the area under the receiver operating characteristic curve (AUROC) and F1-score for each method using the ROCR package (version 1.0–11). DKNN, variance, and dispersion values served as scoring metrics for gene ranking in AUROC calculations.

To evaluate the ability of SciGeneX and singleCellHaystack (version 1.0.2) to generate relevant gene modules, we computed Jaccard index between the simulated gene modules in artificial datasets and the predicted ones. For each true gene module, we selected the top Jaccard index from the predicted modules and computed their median.

Tabula Muris datasets. The same methods were applied with slight parameter variations for the Tabula Muris datasets. In the same way as the artificial datasets, genes expressed in at least four cells were retained for the analysis. We selected genes with SciGeneX using the select_genes() function with parameters : distance_method=“pearson,” noise_level = 0.05, and an FDR of 1e-8. For the closest_neighborhood method, we employed 30 neighbors, while for the reciprocal_neighborhood method, we used 100 neighbors. Graphs were generated with gene_clustering() function by establishing edges for the five closest neighbors (S) of each gene using the closest_neighborhood method. Conversely, the reciprocal_neighborhood method assigns edges a value of zero for genes that do not fall within the neighborhood of their own neighbors. Subsequently, the graph partitioning was performed using an inflation parameter of 1.2. Co-expression modules with <10 genes were then filtered out using the filter_cluster_size() method. For these datasets, modules containing fewer than five cells expressing <35% were excluded using the filter_nb_supporting_cells() method. Finally, the methods used for comparison (DISP, VST, sctransform, M3Drop, singleCellHaystack, and BigSur) were applied with the same functions and versions as in the artificial datasets section. Subsequently, we calculated the AUROC and F1-score for each method in the Tabula Muris datasets using the ROCR package. DKNN, variance, and dispersion metrics were used for gene ranking in the AUROC computations.

### PBMC3k dataset

Data preprocessing. The preprocessing step was performed with the Seurat R package (version 4.3.0). The CreateSeuratObject() method was employed to remove genes expressed in less than three cells as well as cells expressing <200 genes. An additional filter was applied to filter out cells expressing >5% of mitochondrial genes computed with the PercentageFeatureSet() method. Counts were then normalized using the NormalizeData() method with a scale factor of 10 000.

SciGeneX analysis. Genes were selected using the select_genes() method with the normalized count matrix as input. The following parameters were used: distance_method=“pearson,” noise_level = 0.05, and an FDR of 1e-8. The subsequent genes were clustered into co-expression modules using the gene_clustering() function with the closest_neighborhood method. The graph was constructed with three nearest neighbors (S) for each gene and an inflation set to 1.8. The co-expression modules were then filtered to keep the ones containing at least six genes using the filter_cluster_size() method and the ones supported by at least two cells expressing >60% of genes in the co-expression modules using the filter_nb_supporting_cells() method. The top 20 highly co-expressed genes were selected using top_genes() method, and heatmap of their expression were generated using the plot_heatmap() method in which cells were ordered by hierarchical clustering algorithm with ward.D as link.

UMAP visualization. A total of 2000 variable genes were identified using the FindVariableFeatures(). The data were scaled using the ScaleData() method with the genes identified by SciGeneX as “features” parameter. We then performed PCA using the RunPCA() function to generate 50 PCs, which were subsequently used to create the UMAP visualization with the RunUMAP() function.

GO enrichment analysis. GO enrichment analyses based on biological process terms were performed for each co-expression module using the enrich_go() method from the SciGeneX R package. Dotplot visualizations were generated using viz_enrich() and plot_clust_enrcihments() methods.

AUCell analysis. The AUCell R package (version 1.16.0) was used to highlight the fractions of enriched cells for each co-expression module. The ranking of cells was performed with the AUCell_buildRankings() method, and AUC for each co-expression module in each cell was computed with the AUCell_calcAUC() method. Default parameters were used for both methods.

Kmeans clustering. Rare cell populations in Fig. 5 were identified using the k-means clustering algorithm implemented in the amap R package (version 0.8–19). For co-expression module M08 and M11, euclidean distances were computed while for M15, manhattan distance was used.

ImmuNexUT analysis. We used the immune cell gene expression atlas, ImmuNexUT, to retrieve the immune cell types expressing specific co-expression modules in the PBMC3k dataset. This data contains 27 immune cell types (e.g plasmablasts and plasmacytoid dendritic cells) with their respective CPM (counts per million) values for x genes. CPM values were standardized using Z-score standardization and displayed for each gene in co-expression module M11 and M15 for every cell population present in ImmuNexUT database.

Cell cycle regression. As the co-expression module M08 was identified to be related to cell proliferation process, we used those genes to mitigate the effects of cell cycle heterogeneity in the PBMC3k dataset. Cell cycle regression was performed with the Seurat R package (version 4.3.0). A cell cycle score was first computed for each cell using the CellCycleScoring() method and was regressed out with the ScaleData() method. PCA was then performed using the informative genes identified by SciGeneX, and the first 50 PCs were considered the UMAP dimension reduction representation.

### Human T cell trajectory dataset

The processed Human T cell trajectory dataset was downloaded on https://developmental.cellatlas.io website. Genes expressed in >100 cells were retained for the analysis. We used the normalized count provided to first identify co-expressed genes using select_genes() with parameters: distance_method=”pearson”, noise_level = 0.25 and FDR = 5e-4. The identified co-expressed genes are then clustered into co-expression gene modules using the gene_clustering() function with the reciprocal_neighborhood method and an inflation of 2. The subsequent gene modules were filtered to retain the ones containing more than seven genes and with a minimal standard deviation of 0.3. Finally, we used the AUCell R package (version 1.16.0) to highlight the fractions of enriched cells for each co-expression module. Cells were ranked using the AUCell_buildRankings() function, and AUC for each co-expression module in every cell was determined using the AUCell_calcAUC() function. We used the default parameters for both methods.

### Human thymic tissue section dataset

We used a public Visium STs dataset of a human thymic section (from GEO Serie GSE207205) [30]. The preprocessing steps were performed using the Seurat package (version 4.3.0). The count matrix was log normalized using the NormalizeData function, and genes were scaled and centered using the ScaleData function. A set of 2000 highly variable genes was selected with the FindVariableFeatures() function using the VST selection method and used for the PCA. Spots were clustered based on the first 20 PCs using the FindNeighbors() and FindClusters().

SciGeneX analysis. Genes expressed in at least five spots were kept for the analysis. Genes selection was performed on the normalized count matrix with the select_genes() function used with the following parameters: distance_method=“pearson” and row_sum = 5, removing genes expressed in fewer than five spots. The gene_clustering() function was then called with the closest_neighborhood method and parameter set to: distance_method=”pearson” and inflation = 2.2. Finally, those modules were selected to contain at least seven genes and with a minimal standard deviation of 0.12. To quantify the expression level of each gene module, we computed module scores for each spot using the AddModuleScore() function from the Seurat package.

## Results

### Overview of the SciGeneX pipeline

The SciGeneX algorithm operates through a sequence of four key steps: (i) elimination of genes that do not co-express with other genes (Fig. 1A), (ii) construction of a gene’s neighborhood graph (Fig. 1B), (iii) identification of modules of co-expressed genes (Fig. 1C), and (iv) filtering of modules of co-expressed genes (Fig. 1D).

In the initial phase, it employs a density-based filtering (DBF) approach, removing genes that do not exhibit significant co-expression with their neighbors across cells (Supplementary Fig. S1A). This is achieved by comparing the gene–gene distance matrix, often based on the Pearson correlation coefficient, to a null hypothesis of random gene distribution. The distances to the Kth nearest neighbors (DKNN) are computed, and a threshold is determined to distinguish co-regulated genes (see “Materials and methods” section). In the second step, the algorithm constructs a gene’s neighborhood graph using two available methods: “closest_neighborhood” and “reciprocal_neighborhood.” The former establishes edges based on the size (S) of the closest neighborhood of each gene, while the latter considers reciprocal relationships within a neighborhood of size K. Finally, the algorithm employs the MCL to partition co-expressed genes into modules (Supplementary Fig. S1B and C). MCL simulates a random walk through the graph, identifying densely connected regions that represent distinct biological programs. A subsequent filtering step is applied to handle singleton clusters and ensure the reliability of the co-expression modules generated by the algorithm.

### The gene filtering step of SciGeneX outperforms existing feature selection methods

**Figure 2.**
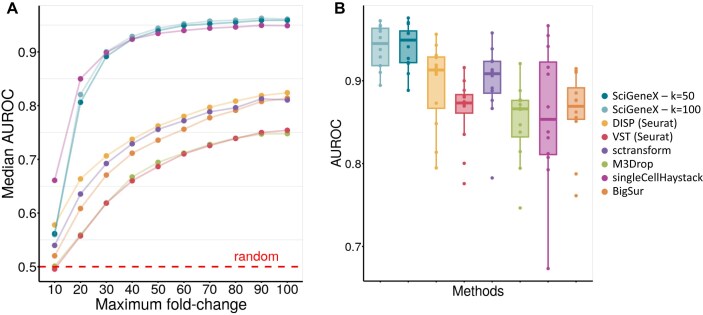
Performance evaluation for finding true DEGs in artificial and experimental datasets. Performances of the neighborhood analysis of SciGeneX were evaluated with AUROC using both artificial and experimental datasets. Two neighborhood sizes (*K*) were used, *K* = 50 (dark blue) and *K* = 100 (light blue). These results were compared with six methods, DISP (yellow) and VST (red) from the Seurat R package, sctransform (purple), M3Drop (green), singleCellHaystack (pink), and BigSur (orange) (**A**) Scatter plots displaying median AUROC computed across artificial datasets. The medians were based on 10 replicates over a maximum fold-change range (10–100, in increments of 10). (**B**) Scatter plots showing AUROC computed across a set of experimental datasets from the Tabula Muris consortium generated on 12 tissues.

To assess the accuracy of the DKNN metric used by SciGeneX in ranking genes, we tested SciGeneX on 100 artificial datasets (Fig. 2A). We generated these artificial datasets using SPARSim [31], incorporating noise and a predetermined number of DEGs distributed into four gene modules. To simulate DEG under various conditions, we used a range of maximum fold-change values between 10 and 100. As shown in Supplementary Fig. S2, a maximum fold change above 10 led to increasingly distinct cell populations. For each maximum fold-change value, we generated 10 independent datasets, resulting in a total of 100 datasets. Finally, we compared the ranking score of SciGeneX with six existing methods: DISP (dispersion) [32], VST (variance-stabilizing transformation) [33], and sctransform [34] from the Seurat package [9] as well as M3Drop [35], singleCellHaystack [36], and BigSur [37].

Using the AUROC curve, we evaluated the ability of the SciGeneX neighborhood analysis to rank DEGs. We set the neighborhood size (*K*) to 50 and 100 and compared SciGeneX to six other methods. The neighborhood analysis on artificial datasets displayed higher AUROC medians compared to all the other methods throughout almost all artificial datasets (Fig. 2A). Only singleCellHaystack showed comparable median AUROC values, but only in artificial datasets with low maximum fold change (10–30). Notably, a maximum fold change of 10 results in poor cell population segregation (Supplementary Fig. S2), leading to expected lower performance.

While artificial datasets are particularly useful to set up a method, they may not accurately reflect experimental dataset counts. In contrast, experimental datasets lack predetermined knowledge of which genes are truly informative (i.e. that differ from noise). To overcome this limitation, we used the well-characterized Tabula Muris [3] datasets and considered the cell population markers identified through DEGs analysis as the true informative genes out of the whole gene set (Fig. 2B). Since the DEG list may differ according to the approach, we used four popular methods including Wilcoxon (Fig. 2B), bimod [38], MAST [39], and *t*-test (Supplementary Fig. S3). DEGs were selected with a minimum log2 fold change of 0.5 (i.e linear fold change of ∼1.4) and a maximum adjusted *p*-value of .05. Similar to the AUROC scoring for artificial datasets, SciGeneX achieved higher AUROC values for experimental datasets when compared to the six other methods (Fig. 2B).

The DKNN metrics employed by SciGeneX outperform existing methods in accurately ranking informative genes using both artificial and experimental datasets. This makes it a valuable method for feature selection.

### The gene partitioning step of SciGeneX outperforms existing gene module methods

**Figure 3.**
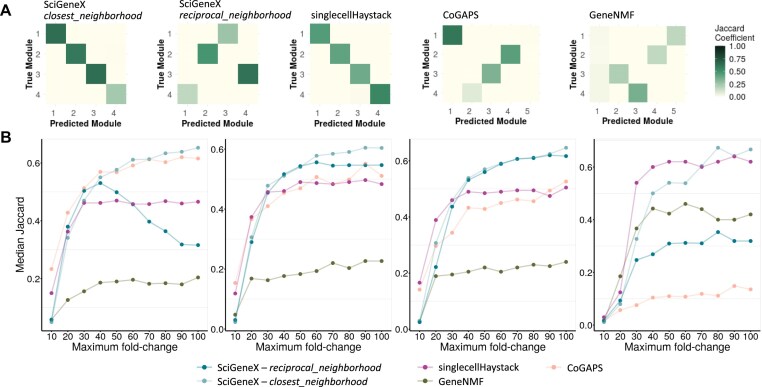
Evaluation of the ability to generate relevant gene modules. (**A**) A pairwise Jaccard index heatmap to visualize the relationship between the true gene modules and the gene modules generated by the closest_neighborhood and reciprocal_neighborhood approaches of SciGeneX and by singleCellHaystack, CoGaps, and GeneNMF (from left to right). (**B**) Scatter plots showing the median of the top Jaccard index computed for four gene modules simulated across a set of artificial datasets with 10 replicates per maximum fold change (10–100, in increments of 10).

In addition to feature selection, SciGeneX also provides a gene partitioning step based on the MCL algorithm. To evaluate the ability of SciGeneX to generate relevant gene modules, we measured the pairwise Jaccard index between the gene modules generated from artificial datasets and those discovered by SciGeneX. An example of these results is represented in Fig. 3A. For each true gene module, we identified the highest Jaccard index among the predicted ones and computed their median. We also compared these results with methods designed to generate gene modules, singleCellHaystack, CoGAPS, and GeneNMF.

Overall, across the four simulated gene modules, the closest_neighborhood approach showed superior or similar performances compared to the other methods (Fig. 3B). The reciprocal_neighborhood approach shows more nuanced results with higher performances observed for modules 2 and 3 and lower performance for modules 1 and 4. We consistently found that GeneNMF tends to form modules of limited sizes (Supplementary Fig. S4), resulting in poor performance as highlighted by rather low Jaccard index values, whereas CoGAPS performances decrease when true module size decreases. The singlecellHaystack method showed more variable results compared to the SciGeneX closest_neighborhood approach.

Altogether, SciGeneX demonstrated an ability to generate relevant gene modules, and the closest_neighborhood approach outperforms singleCellHaystack in accurately retrieving simulated gene modules.

### Combination of co-expressed gene modules reveals cell populations

One of the key advantages of the SciGeneX algorithm is its ability to partition informative genes into co-expression modules. To evaluate its ability to produce gene modules from selected informative genes that would overlap with specific biological functions, we used the PBMC3k dataset [24] as a gold standard. When employing the standard Seurat tutorial, cells were classified into nine reference cell populations. The SciGeneX neighborhood analysis identified 3340 genes partitioned into 377 co-expression modules. After filtering, a set of 977 informative genes clustered into 16 co-expression modules was generated. Using these genes, we generated UMAP visualizations of the cells (Supplementary Fig. S5A–C) and computed silhouette scores (Supplementary Fig. S5D), a metric that quantifies cluster cohesion and separation. Our results showed that SciGeneX achieved the highest silhouette score compared to Seurat, indicating improved resolution (Supplementary Fig. S5).

**Figure 4.**
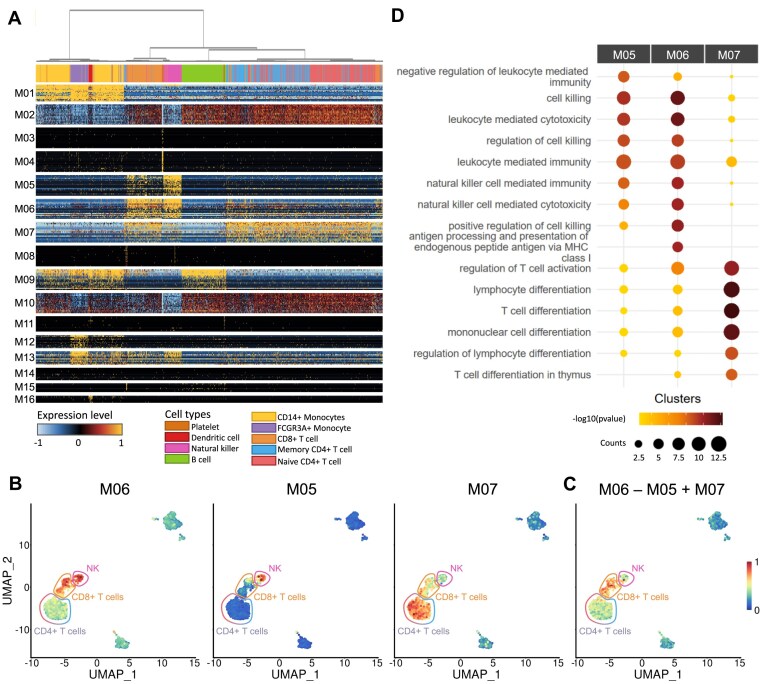
Combinations of gene modules generated by the SciGeneX algorithm reveal cell populations in relation to biological processes. (**A**) Heatmap displaying normalized expression levels of the top 20 genes within each co-expression module generated by the SciGeneX algorithm. Cells have been ordered using a hierarchical clustering algorithm. The labels of the cell populations, as defined in the original Seurat results, are shown on the top of the heatmap. (**B**) UMAP plot of PBMCs colored by their AUCell score for modules M06, M05, and M07 from left to right. (**C**) The AUCell scores of module M06 were subtracted from those of module M05 and added to those of module M07 to obtain a composite score, which is plotted on the UMAP. (**D**) Dotplot showing the GO enrichment analysis for gene modules M05, M06, and M07. The top five enriched GO terms are displayed for each gene module, providing insights into the biological processes associated with the previously mentioned co-expression modules.

To ensure that gene modules reflect known cell populations, we applied a hierarchical clustering algorithm to order the cells, which successfully highlighted the previously identified cell populations (Fig. 4A). As an example, module M04 was found to be specifically active in a previously characterized platelet population, while module M16 demonstrated specific expression in dendritic cells (Fig. 4A). Additionally, several modules demonstrated shared expression among multiple cell populations. For instance, module M05 exhibited high expression in natural killer cells and moderate expression in CD8 + T lymphocytes. Module M06 displayed a high level of expression in both populations. On the other hand, module M07 showed predominant expression in T lymphocyte populations, including CD8 + and CD4 + T lymphocytes (Fig. 4A).

As cell populations are the result of differentiation processes that lead to the activation or repression of specific groups of genes, cell types and states can be viewed as combinatorics of activated and repressed pathways. We hypothesized that these repressed/activated pathways could be mimicked by combinations of gene module activities that would reveal underlying cell types. To summarize module activity, we computed AUCell scores for each of them across all cells and projected them on a UMAP. We can visualize individual module activities (i.e. AUCell scores) (Fig. 4B) and sum up the activities of shared modules M06 and M07 subtracted by the activity of shared module M05 (Fig. 4C). This can be interpreted as looking for cells with high levels of modules M06 and M07 while excluding cells with high levels of module M05. Interestingly, such a combination revealed a new module with high specificity in CD8 + T lymphocytes, which was supported by functional enrichment analysis (Fig. 4D). Indeed, module M07 exhibits enrichment in biological processes related to T cell differentiation, a function shared between CD4 + and CD8 + T cells. Module M06 was found to be enriched in genes related to cell killing and cytotoxicity, which are functions shared between CD8 + T cells and NK cells. While the functional enrichment of module M05 closely resembled that of module M06, a more detailed examination of the gene list revealed a significant presence of NK cell-specific markers including various killer cell immunoglobulin like receptor (such as IR2DL3 and KIR3DL2) and killer cell lectin like (including KLRC1, KLRD1, and KLRF1).

Altogether, these results demonstrate the ability of SciGeneX to capture co-expressed gene modules associated with both specific and shared expression between cell populations. Gene modules are almost all significantly associated with specific terms from biological processes (Supplementary Fig. S6). We show that these modules can be used alone or combined to reflect cell population heterogeneity and assist downstream cell clustering analysis.

### Co-expression modules reveal rare cell populations

**Figure 5.**
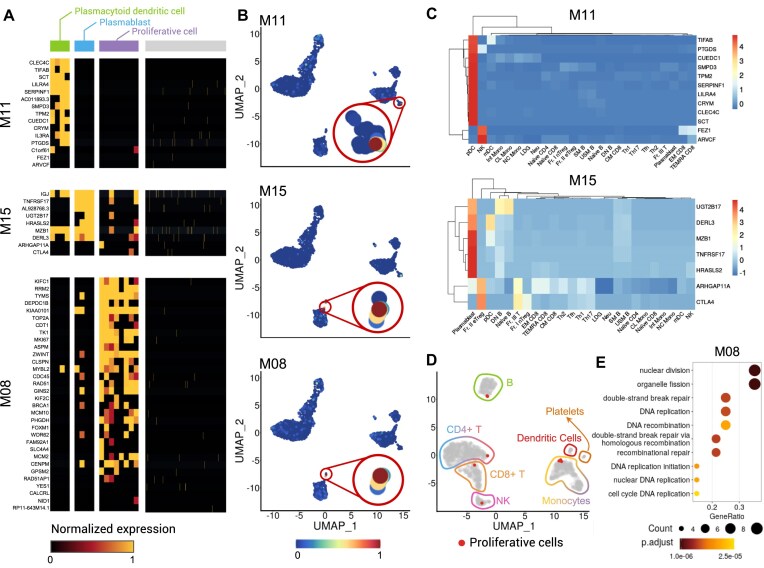
Identification of rare cell populations using co-expression modules. (**A**) Heatmap representing normalized expression levels within co-expression modules M08, M11, and M15 generated by the SciGeneX algorithm. Cells have been partitioned using K-means clustering method. The cell clusters are indicated at the top of the heatmap with four cells in green, four cells in blue, eight cells in purple, and 2622 in gray. (**B**) UMAP plot of PBMCs colored by their AUCell score for co-expression modules M08, M11, and M15 (top to bottom). (**C**) Heatmap displaying the CPM values for immune cell types from ImmuNexUT, focusing on genes from co-expression modules M11 and M15. (**D**) UMAP representation of PBMCs with gene expression regressed for genes belonging to co-expression module M08. Proliferative cells are highlighted in red. (**E**) Dotplot showing the results of GO enrichment analysis for gene module 8. The top five enriched GO terms are displayed, providing insights into the biological processes.

While the original analysis on the PBMC3k dataset revealed nine cell populations, the gene modules revealed by SciGeneX suggested a more intricate situation. Close inspection of the hierarchical clustering in Fig. 4A revealed that the expression of modules M11 and M15 was restricted to two different and yet small cell groups. Applying a two-class k-means clustering to the expression data of module M11 and M15 elucidated these populations (Fig. 5A). In particular, cells expressing genes from modules M11 and M15 are clustered in specific areas of the UMAP (Fig. 5B). To further characterize these populations, we interrogated the ImmuNexUT database to identify cell type using genes from co-expression modules M11 and M15. Genes from module M11 were predominantly expressed in plasmacytoid dendritic cells (Fig. 5C), consistent with markers LILRA4, CLEC4C, SERPINF1, and ILR3A also reported in the PanglaoDB database [40]. Similarly, genes in module M15, including TNFRSF17, UGT2B17, and MZB1, were mainly active in plasmablasts previously reported as markers of this population.

Moreover, using a hierarchical clustering analysis, we identified genes from module M08 (Fig. 5A) that were expressed in distantly related populations (Fig. 4A). A two-class k-means clustering was also used to reveal these populations. These cells appeared to be co-localized in the UMAP (Fig. 5B). GO analysis unveiled that genes from module M08 were related to cell proliferation (Fig. 5E). We thus regressed expression data with genes from module M08 and performed a new gene reduction and UMAP representation. Contrary to what was initially thought, these cells did not cluster exclusively in one population but distributed among several ones. This highlighted very tiny sub-populations of proliferating natural killer cells, CD14 + monocytes, and CD4 + T lymphocytes (Fig. 5D).

Our results showcase the ability of the SciGeneX algorithm to reveal extremely rare yet functionally relevant cell populations or cell states not yet characterized using a dataset that has already been extensively explored.

### Gene modules reveal cellular heterogeneity in a continuum of differentiating T cells

**Figure 6.**
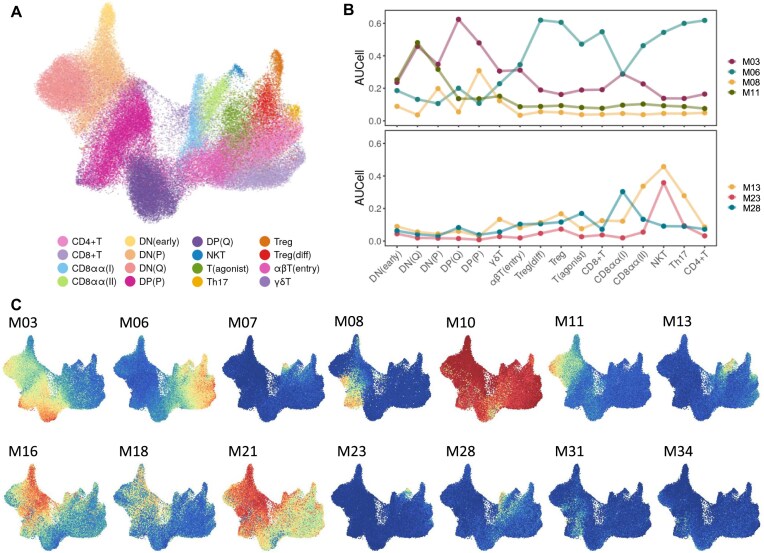
Co-expression modules reveal differentiation processes in a continuum of developing T cells. (**A**) UMAP plot of T cell differentiation stages from human thymus. Cells are colored by the cell types defined in the original study. (**B**) Evolution of the expression of gene modules across the differentiation of T cells. The top graph shows modules M03, M06, M08, and M11 related to cellular functions, and the bottom graph shows the modules M13, M23, and M28 related to known differentiation stages. (**C**) UMAP plot colored by AUCell scores for co-expression modules found by SciGeneX.

The PBMC3k dataset previously analyzed was characterized by a low complexity and the presence of terminally differentiated cells. We directed our focus toward a substantially larger dataset comprising developing cells. To this end, we used the “Human T cell trajectory” dataset [25] containing 76 994 cells (∼30 times more than the PBMC3k dataset) that were classified into 16 main cell populations (Fig. 6A), offering a continuous spectrum of differentiating cells. We identified a total of 2821 informative genes, distributed across 71 modules (Fig. 6C and Supplementary Fig. S7) using the reciprocal_neighborhood method (See Supp_file_Gene_Modules_Thymus_Single_Cell.xls).

Gene modules reflected the diverse stages characterizing thymic T-cell development. Module M11 showed a specific expression in early T-cell development (Fig. 6B) confirmed by the presence of key factors of very early T-cell development, including NOTCH1 and HES1 [41]. While module M03 is more specific to the double-positive stage (Fig. 6B) with prominent markers such as CD8A, CD8B, and CD4, in addition to the VDJ recombination actors RAG1 and RAG2 [42]. Module M06, in turn, contains more mature T-cell markers (Fig. 6B), including CD5 and CD28, accompanied by interferon-inducible genes such as IFI44L, IFITM1, HLA-A, HLA-B, and IRF7.

Notably, gene modules closely mirrored three recently identified CD8aa + T cell populations: GNG4 + CD8aa + T(I), ZNF683 + CD8aa + T(II), and EOMES + CD8aa + NKT-like [25]. Indeed, module M28, specific to GNG4 + CD8aa + T(I) cells, include three known markers, GNG4, CREB3L3, and PDCD1, as well as additional genes such as NFATC1, FZD3, CD200, and CD82. Interestingly, module M07 was even more specific to this population with gene markers including XCL1, XCL2, and CD72. Module M13 reflected the ZNF683 + CD8aa + T(II) cell population characterized by a mixed αβ and γδ lineage[29]. It revealed markers associated with the γδ T-cell receptors (TRDV1, TRDV2) alongside the marker CD84 and various killer cell lectin-like and killer cell immunoglobulin-like receptors (KIR3DL2, KLRB1). Finally, module M23 was found to be specific to the EOMES + CD8aa + NKT-like population exhibiting specific surface markers (CD160, FCGR3A, IL18RAP, KLRD1, and FASLG) and demonstrated the production of several cytokines, including CCL3, CCL3L3, and IFNG (Fig. 6C).

Gene modules were also found to be specific to regulation of several biological processes that govern T-cell differentiation. Module M08 corresponded to a proliferative state (MKI67, TOP2A), module M10 reflected protein synthesis (RPL10, RPL11), while modules M16, M18, and M21 reflected the developmentally regulated usage of various genes related to oxidative phosphorylation (COX17, COX8A, NDUFA1B). Moreover, T-cell differentiation is marked by profound chromatin rearrangements particularly highlighted by module M31 containing 20 genes related to histones or module M34 composed also of 18 genes coding for histones that harbor a more restricted expression program (Fig. 6C).

Altogether, SciGeneX shows its capacity to create highly biologically meaningful gene modules that depict the complex processes underlying T-cell differentiation, providing valuable insights for a more refined population characterization.

### Scigenex applied to STs data allow in depth resolution of tissue molecular architecture

**Figure 7.**
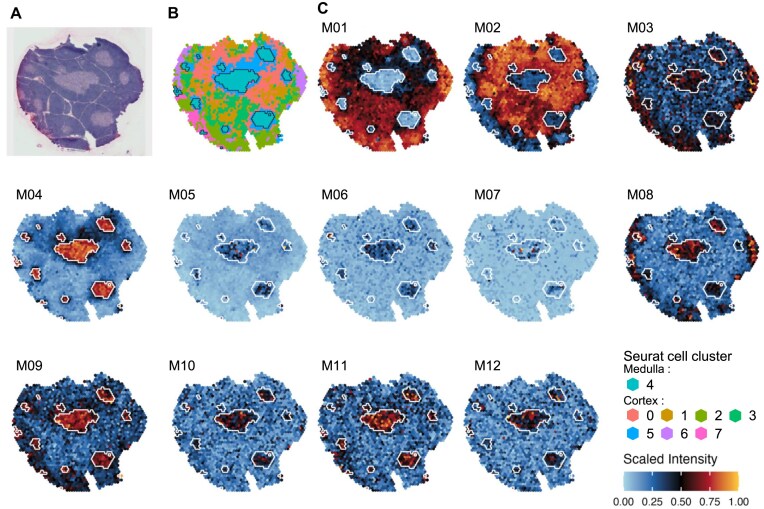
Co-expression modules generated by the SciGeneX algorithm on STs of a human thymus section. (**A**) H&E image of human thymus and matching spots annotated into eight topological areas. Medullary areas appear as internal zones with less intense labeling. (**B**) Spot clusters as identified by the FindClusters Seurat method (default parameters). The regions surrounded by a thin black line corresponds to the Seurat spot cluster associated with the medulla (Seurat spot cluster #4 in legend). (**C**) Scaled intensity per spot for 12 co-expression modules generated by the SciGeneX algorithm. To ease comparison, the Seurat spot cluster #4 is also depicted with a thin white line in all panels from (**C**).

To assess the ability of SciGeneX in detecting co-expression modules in ST data, we ran SciGeneX on a recently published Visium dataset interrogating the transcriptome of a human thymus section [30]. In this study, the authors previously stained thymic slices using H&E to delineate the two primary regions within the thymus: the cortex situated in the organ’s outer layer and hosting early T-cell precursors, and the medulla, which contains more mature thymocytes (Fig. 7A). The Seurat reference analysis identified seven spot clusters (Fig. 7B) and corresponding DEG.

Using Scigenex a set of 21 gene modules (containing at least 8 genes) corresponding to a total of 1402 genes (mean number of genes per module: 70.1) were revealed (Supplementary Fig. S8 and Supp_file_Gene_Modules_Thymus_Spatial_Transcriptomics.xls). The spatial distribution of these co-expression modules was highly concordant with the known architecture of the thymus. As an example, modules M01 and M02 were found to be highly expressed in the outer cortex. Surprisingly, while the medulla was represented by a unique spot cluster in Seurat analysis (spot cluster #4), Scigenex was able to reveal a far more complex scenario. Indeed, numerous modules (Fig. 7C and Supplementary Fig. S9) with spatially restricted patterns in the medulla of with expression shared between the cortex and medulla were found (e.g. M3–M14, M16–M21). Most of the gene modules could be unambiguously associated with functions known to be active in the thymus (Supplementary Fig. S10). This encompasses GO terms associated with T-cell development “V(D)J recombination and alpha-beta T-cell activation” (M01), “positive regulation of lymphocyte activation” (M04), “Chemokine activity” (M06), “response to interferon—alpha” (M11), or “ribosome assembly” (M15). Additionally, our method was sensitive enough to detect several rare populations of highly specialized thymic epithelial cells (TECs) including: muscle mimetic medullary TEC (M07, enriched for “striated muscle contraction”) known to specifically express skeletal muscle genes (MYOG, TNNT1-3, MYL4…), corneoTEC (M05, enriched for “epidermal cell differentiation”) known to express keratinocytes or corneocytes markers (e.g. DMKN, LY6D, and SPINK5) or AIRE + mTECs (M06, enriched for “CCR chemokine receptor binding”) [43].

Altogether, these results demonstrate that, applied to Visium datasets, SciGeneX algorithm provides a highly resolutive overview of cellular diversity. Our results also indicate that even with a low resolution technology (spot diameter 50 μm), SciGeneX is able to identify spatially variable modules expressed in widespread or very rare mimetic cell populations [44].

## Discussion

Here, we introduced SciGeneX, which proposes an alternative approach revolutionizing the scRNA-seq or ST analysis process by looking at the broader panorama of gene co-expression without prior cell clustering. Using gene neighborhood analysis and graph clustering algorithm, SciGeneX shows an innovative perspective by generating co-expression gene modules, providing a comprehensive overview of the underlying biological processes within scRNA-seq or ST datasets. In contrast to conventional methods, SciGeneX aligns with the Waddington’s model [45], relying on the dynamic interplay of cellular states shaped by the activation or repression of biological pathways. Thus, cellular populations could be characterized by the activation and repression of various pathways, some of which may be specific or shared among different cell types.

In our study, SciGeneX has shown its ability to provide a very rich molecular overview of underlying cell populations. Indeed, it generated co-expression gene modules that collectively mirror well established cell populations. These gene modules can be either shared among multiple populations or selectively expressed in specific ones. Moreover, cellular populations could be characterized by the activation and repression of various pathways. Thus, we showed that a combination of co-expression gene modules is able to highlight specific cell populations. For example, with a combination of three gene modules in the PBMC3k dataset, we were able to specifically highlight a population of CD8 + T cells. Conversely, certain modules can accurately detect the presence of rare undiscovered populations (e.g. plasmacytoid dendritic cells and plasmablasts) expressing highly specific markers. These populations with distinct markers were previously undetected using the Seurat pipeline, highlighting several limitations of conventional approaches.

Another benefit of Scigenex is its potential utility to provide biological cues to assist in accurately defining cell populations within a dataset. Indeed, while Scigenex does not offer a direct solution for cell clustering, it can contribute to decision-making by offering insights into activated or repressed pathways using heatmap visualization and mapping expression of specific or combined gene modules on a UMAP. This is crucial, as inaccurately defined cell populations can result in heterogeneous clusters, leading to reduced statistical power.

SciGeneX also gave very promising and robust results when analyzing ST data (Visium). ST is a rapidly evolving field with several concurrent but also complementary technologies. SciGeneX was able to uncover very rare and recently discovered mimetic mTEC populations in ST dataset [46], which were not described in the original study [30]. As ST continues to establish itself as a standard method, our algorithm could serve as a highly effective approach for investigating cellular diversity within spatial dimensions.

Additional datasets were also tested and SciGeneX consistently uncovered highly relevant gene modules (note that a Visium brain dataset example is also provided in the accompanying vignette of the R package). Nevertheless, due to dataset diversity, it would be impractical to establish a one-size-fits-all set of optimal parameters. While this may seem like a limitation, users have the flexibility to adjust key parameters such as neighborhood size, the inflation parameter of the MCL algorithm, or filtering steps. Regarding filtering steps, we frequently achieved excellent results by simply choosing either the cluster size or standard deviation. However, the package also offers filtering options based on additional criteria, including silhouette similarity score, Dunn index, and connectivity score as proposed by the clValid R package [47]. Moreover, as implemented in the *gene_clustering* function, other graph partitioning algorithms can be applied such as Louvain and Walktrap. SciGeneX also provides two approaches to construct the graph, the *closest_neighborhood* or the *reciprocal_neighborhood*. The choice between these two approaches depends on the desired outcome of the gene modules. The *reciprocal_neighborhood* approach is more sensitive to subtle gene relationships, often generating a larger number of smaller co-expressed modules. In contrast, the *closest_neighborhood* approach incorporates more genes into each module by constructing a proximity-based graph, making it more effective for capturing exhaustive co-expression patterns while tending to be functionally less resolutive.

Interestingly, SciGeneX is also intended to be a more general container of gene modules within the R ecosystem. In this way the ClusterSet object may be created from a Seurat object or from an expression matrix and gene list. This makes it fully compatible with other methods including M3Drop [35], BigSur [37], DUBStepR [28], singleCellHaystack [36], Seurat, COTAN [48], or Hotspot [49]. Once loaded, gene modules obtained from other methods may be manipulated easily by taking advantage of the ClusterSet methods.

In summary, SciGeneX is an approach of choice for unraveling cellular and molecular diversity in the fields of single-cell approaches and ST. Its ability to offer a complete molecular snapshot of cell populations is particularly exciting for researchers keen to uncover hidden information in their data. The tool’s accessibility, adaptability and compatibility with other methodologies makes it a valuable asset in these research fields.

## Supplementary Material

lqaf043_Supplemental_Files

## Data Availability

The SciGeneX R package is available on Github (developmental version) https://github.com/dputhier/scigenex. The 12 10× genomics droplet datasets were downloaded as Seurat objects from the Tabula Muris web site (https://tabula-muris.ds.czbiohub.org). Annotations from the original studies were available in the metadata slot of the Seurat objects. The count matrix of the scRNA-seq PBMC3k dataset was obtained from 10x Genomics web site and can be downloaded from https://cf.10xgenomics.com/samples/cell/pbmc3k/pbmc3k_filtered_gene_bc_matrices.tar.gz. The scRNA-seq of thymic development dataset is available on ArrayExpress using accession number E-MTAB-8581 and was downloaded through the Human Cell Atlas Development portal (https://developmental.cellatlas.io/). The spatial transcriptomics dataset of human thymus was obtained from the Gene Expression Omnibus under accession number GSE207205. Only GSM6281326 was used. Scripts and detailed instructions to reproduce the analysis for this manuscript are available on FigShare (DOI : 10.6084/m9.figshare.26367160). A Docker image is also available on Zenodo (https://zenodo.org/records/12801381) to easily reproduce the analysis.
