# Supplementary material for: SciGeneX: enhancing transcriptional analysis through gene module detection in single-cell and spatial transcriptomics data
Source: NAR Genom Bioinform. 2025 Apr 17;7(2):lqaf043. doi: 10.1093/nargab/lqaf043 (PMC12004220; doi:10.1093/nargab/lqaf043)
Supplement: lqaf043_Supplemental_Files [file lqaf043_supplemental_files.zip › supplementary_data.docx]

**Supplementary Information for:**

**SciGeneX: Enhancing transcriptional analysis through gene module detection in single-cell and spatial transcriptomics data.**

Julie Bavais^1,2^, Jessica Chevallier^1,2^, Lionel Spinelli^1,2^, Serge A. van de Pavert^2*^ and Denis Puthier^3*^

1 Aix-Marseille University, Inserm U1090, Theories and Approaches of Genomic Complexity (TAGC),

Turing Centre for Living systems, Marseille, France.

2 Aix-Marseille Univ, CNRS UMR7280, Inserm U1104, Centre d’Immunologie Marseille Luminy (CIML),

Turing Centre for Living systems, Marseille, France.

3 Aix-Marseille University, Inserm U1090, Theories and Approaches of Genomic Complexity (TAGC),

MarMaRa Institute, Turing Centre for Living systems, Transcriptomics and Genomics Marseille Luminy (TGML), Marseille, France.

**Corresponding authors:** vandepavert@ciml.univ-mrs.fr, denis.puthier@univ-amu.fr

**This PDF file includes:**

Runtime consideration of SciGeneX

Supplementary Figure S1. Application of SciGeneX on the Human PBMC3k Dataset: Concrete Examples of Gene Selection.

Supplementary Figure S2. UMAP representation of artificially generated scRNA-seq datasets.

Supplementary Figure S3. Performance evaluation of different methods to find DEGs in Tabula Muris datasets.

Supplementary Figure S4. Distribution of gene count per module across methods.

Supplementary Figure S5. Comparison of UMAP visualizations based on gene seleted by Seurat and SciGeneX methods.

Supplementary Figure S6. Functional Enrichment Analysis of Co-Expressed Gene Modules Identified by SciGeneX.

Supplementary Figure S7. UMAP visualization of AUCell scores for each gene module generated by SciGeneX in T cells trajectory dataset.

Supplementary Figure S8. Heatmap representation of co-expressed gene modules identified by SciGeneX in spatial transcriptomics of a human thymus section.

Supplementary Figure S9. Scaled expression intensity profiles of additional co-expressed gene modules on spatial transcriptomics dataset of a human thymus section.

Supplementary Figure S10. Network representation of functional enrichment analysis of co-expressed gene modules obtained on spatial transcriptomics dataset of a human thymus section.

**Runtime consideration of SciGeneX**

In terms of performance, running the PBMC3k single-cell experiment dataset on a MacBook Pro with a 2.3 GHz Intel Core i9 takes approximately 3.8 minutes. For the brain Visium dataset available from the SeuratData library (anterior 1 slice), it takes approximately 9 minutes to complete. For the Park dataset (77k cells) it takes about one hour. While time can be a constraint, the most significant limitation lies in memory usage. Running the entire pipeline necessitates a computer with a minimum of 16GB of RAM, and dealing with very large datasets (e.g., tens of thousands of cells) may necessitate the use of a dedicated computing server. Nevertheless, for typical datasets, the analysis can be carried out on a laptop. Finally, it's important to note that the current version of the MCL algorithm implementation is only compatible with Linux and macOS operating systems. Although an R implementation is available, our testing has identified important differences in partitioning results when compared to the original implementation. Exploring the possibility of integrating the MCL algorithm for Windows users in the future could broaden the accessibility and utility of the SciGeneX methodology, making it a more inclusive and versatile analytical tool.


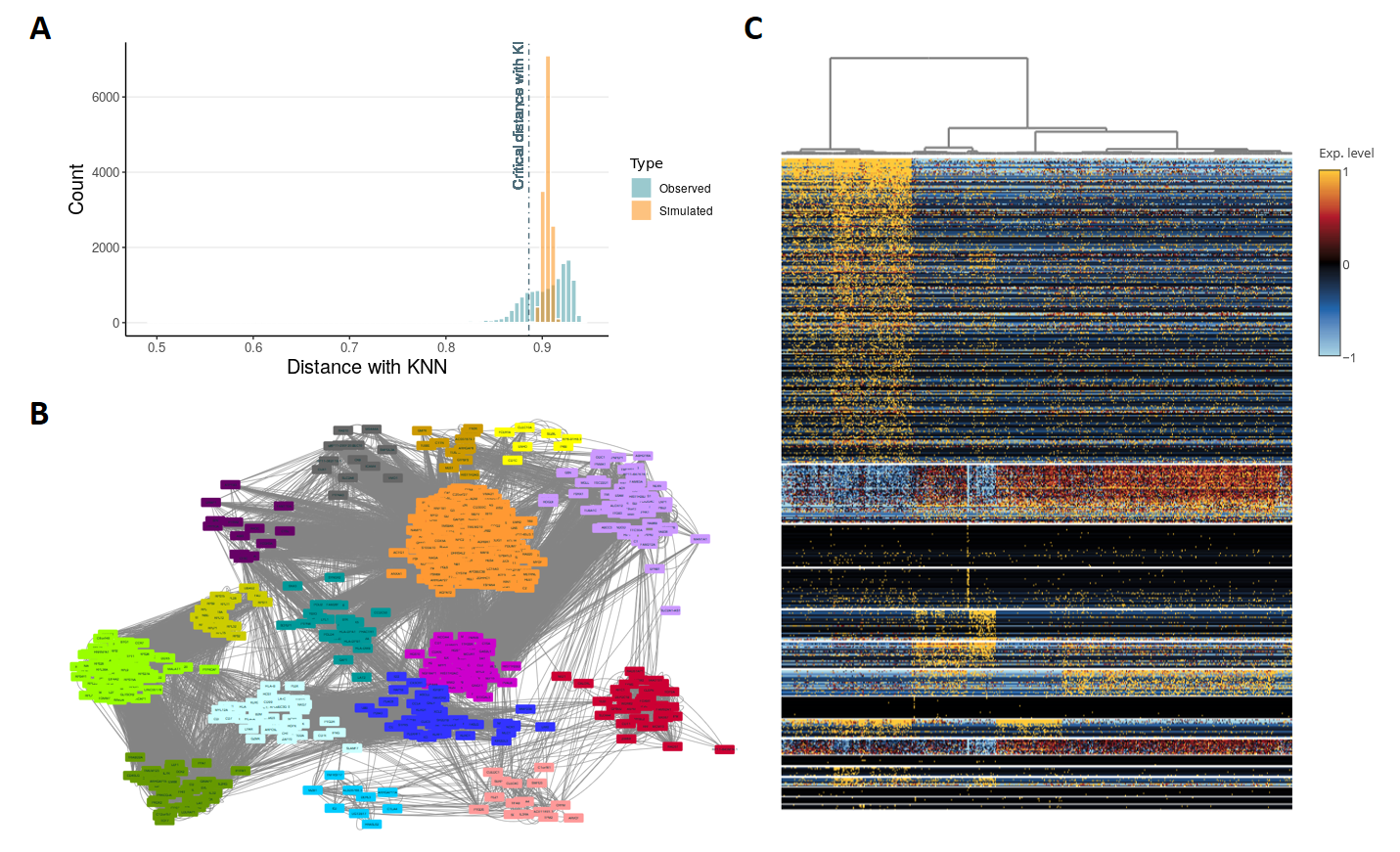


**Figure S1. Application of SciGeneX on the Human PBMC3k Dataset: Concrete Examples of Gene Selection.** (A) Histogram of the distances with KNN (DKNN) for each in the human PBMC3k dataset with observed distances in blue and simulated distances in orange. (B) Graph illustrating the gene modules identified by SciGeneX. Each node represents a gene, with its color indicating the module to which it belongs. Edges represent the DKNN. (C) Heatmap displaying normalized expression levels of the genes within each co-expression module generated by the SciGeneX algorithm. Cells have been ordered using a hierarchical clustering algorithm.


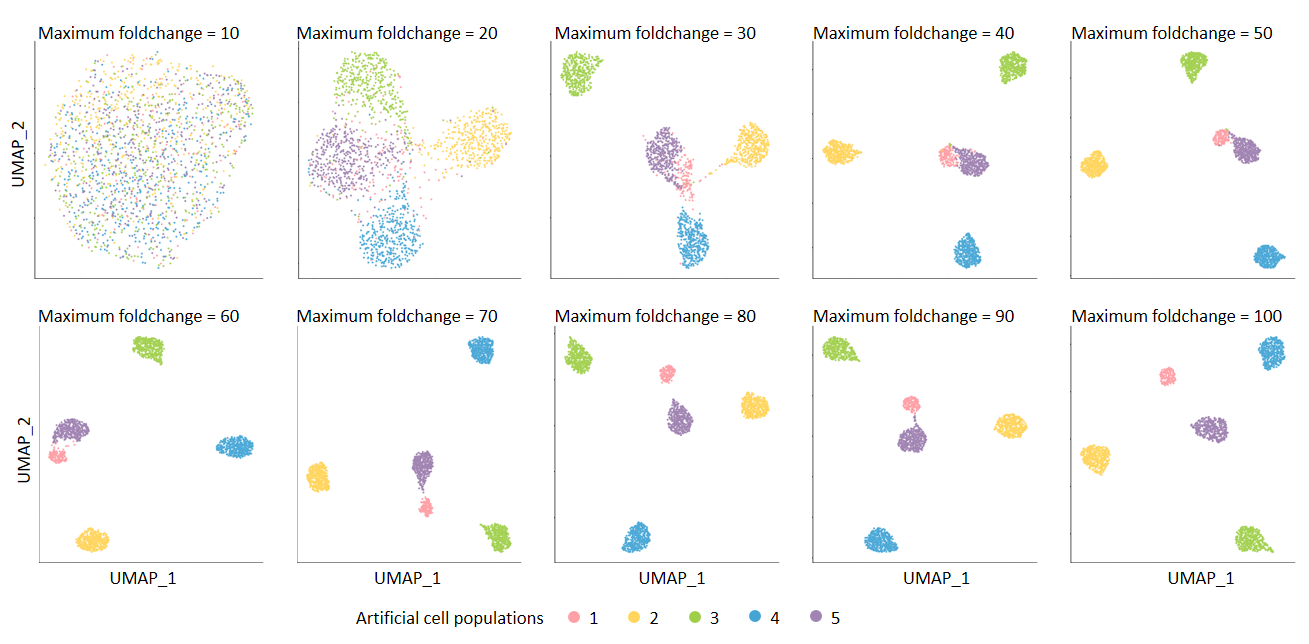


**Figure S2. UMAP representation of artificially generated scRNA-seq datasets.** UMAP representations of 10 artificially generated scRNA-seq datasets in which DEGs were simulated with maximum fold-changes ranging from 10 to 100 in increments of 10. Cell populations are indicated by distinct colors: purple, green, yellow, blue, and pink.

**
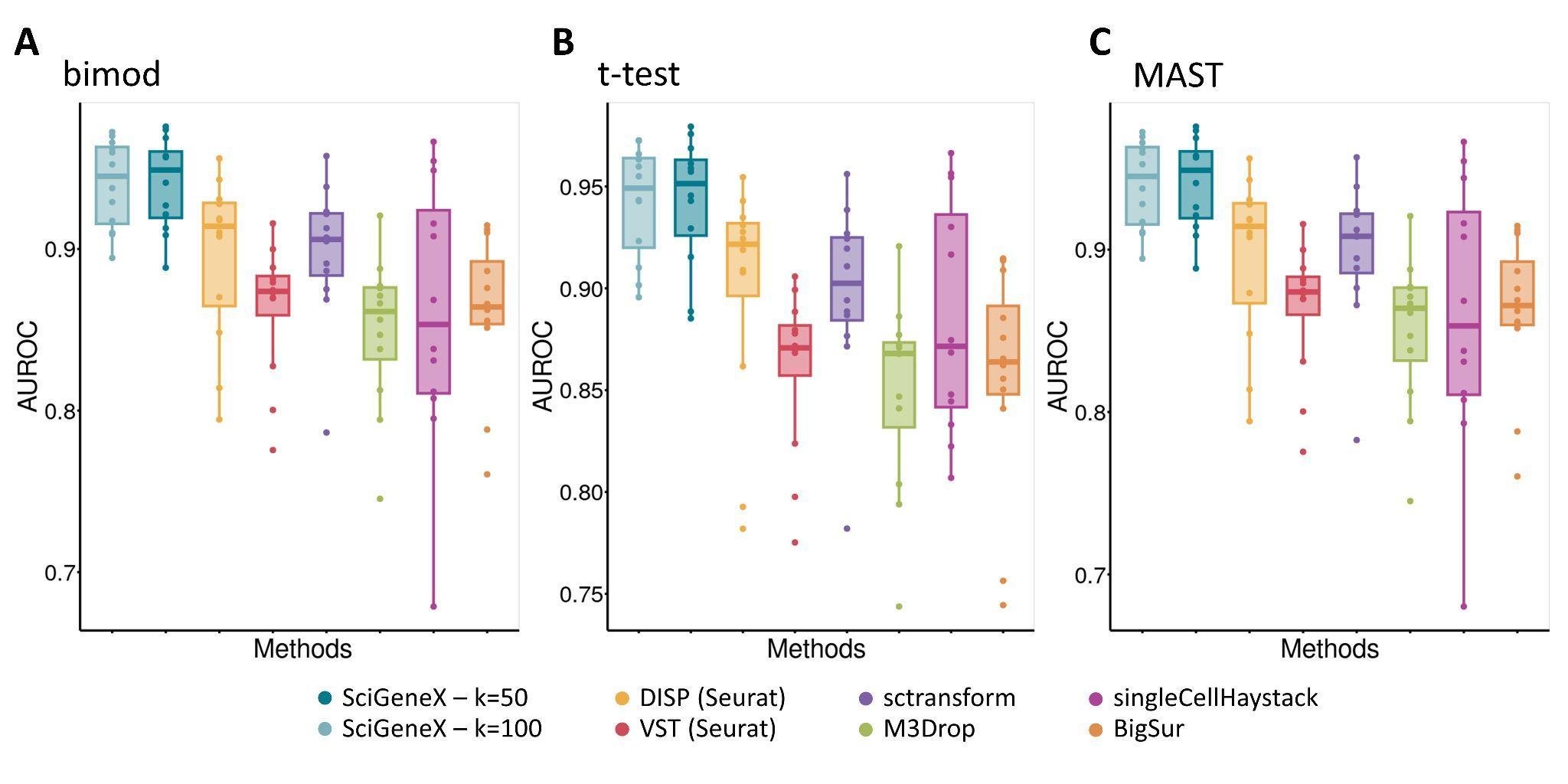
**

**Figure S3. Performance evaluation of different methods to find DEGs in Tabula Muris datasets.** Performances of SciGeneX and six existing methods (DISP, VST, sctransform, M3Drop, singleCellHaystack and BigSur) were computed across a set of experimental datasets from the Tabula Muris consortium generated on 12 tissues : bladder (B), heart and aorta (HA), kidney (K), liver (Li), limb muscle (LM), lung (Lu), marrow (M) mammary gland (MG), spleen (S), thymus (Th), tongue (To) and trachea (Tr). True DEGs have been defined using three methods, bimod (A), t-test (B) and MAST © and AUROC was computed to evaluate the performances of SciGeneX neighborhood analysis for two k values, k=50 (dark blue) and k=100 (light blue) and the six other methods.


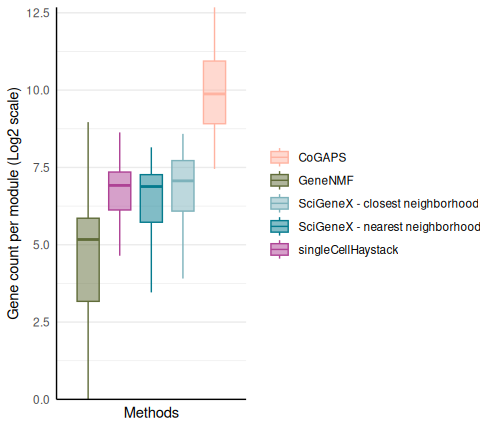


**Figure S4. Distribution of gene count per module across methods.** Boxplots representing the log2-transformed gene count per module across five different computational methods: GeneNMF (green), SciGeneX closest neighborhood (light blue), SciGeneX nearest neighborhood (dark blue), singleCellHaystack (purple), and CoGAPS (orange). Each boxplot displays the median, interquartile range (IQR), and whiskers extending to 1.5× the IQR.


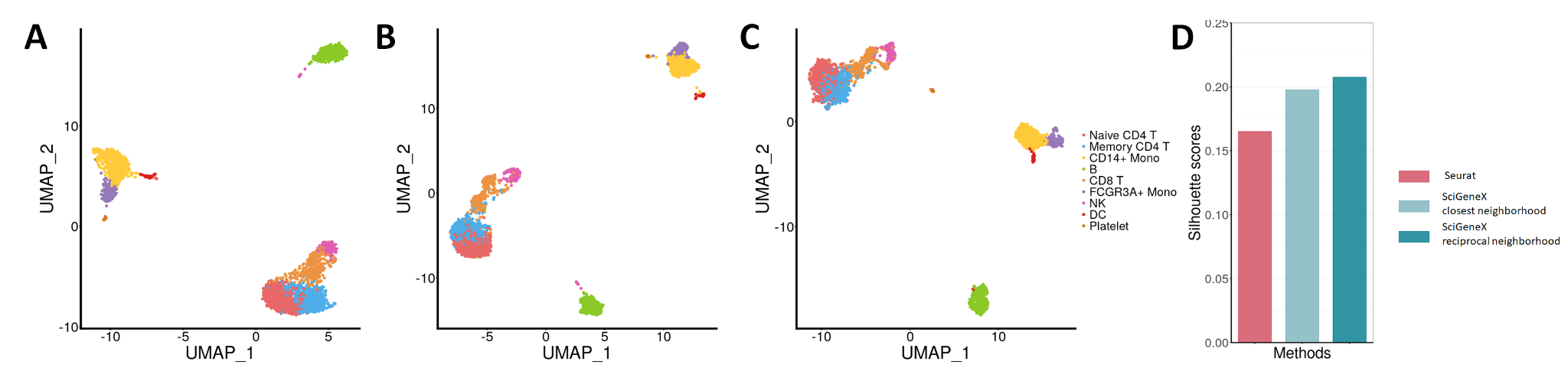


**Figure S5. Comparison of UMAP visualizations based on genes selected by Seurat and SciGeneX methods.** Each panel displays the UMAP visualization of PBMC3k dataset with colors corresponding to the cell populations as initially described in the dataset annotations. The left panel shows the UMAP generated with genes selected by Seurat (A) reference pipeline, while the middle and right panels use genes identified by the SciGeneX methods, *closest_neighborhood* (B) and *reciprocal_neighborhood* (C). To obtain (B) and (C) the list of genes selected by SciGeneX were passed to the Seurat *ScaleData()* function (argument *features*). In (B) and (C) several subpopulations tend to better segregate in the CD8+ T cell population. In the same way, NK cells tend to better discriminate. (D) Barplot showing the average silhouette scores for cell clusters. Each bar represents the mean silhouette score of a method among Seurat and the two SciGeneX methods (closest neighborhood, reciprocal neighborhood).


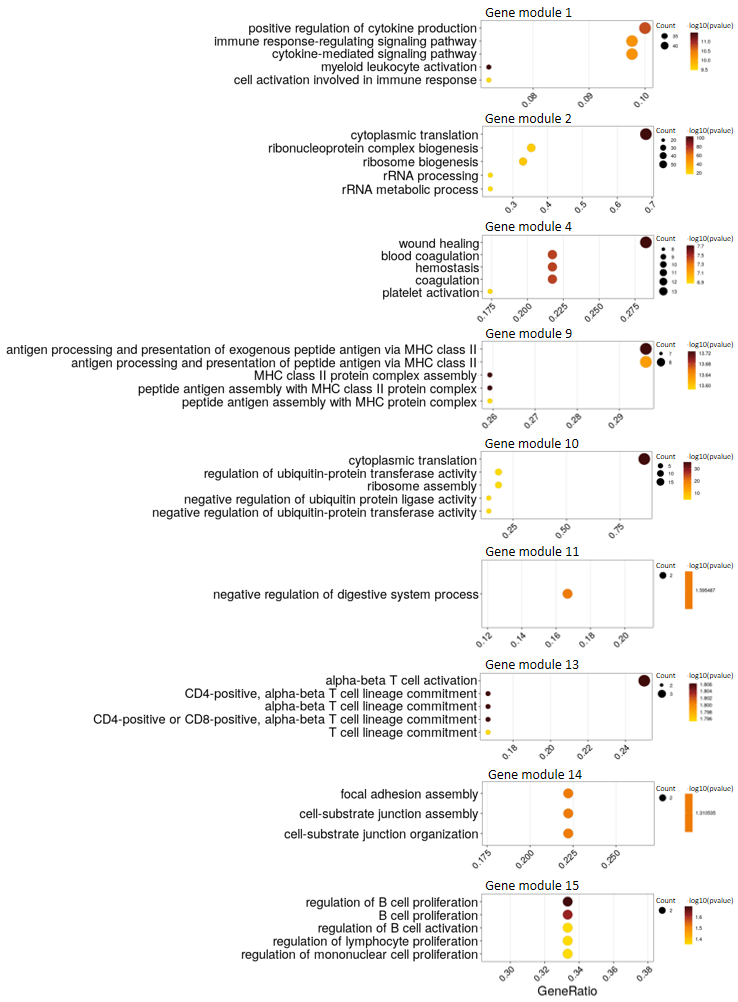


**Figure S6. Functional Enrichment Analysis of Co-Expressed Gene Modules Identified by SciGeneX.** Dotplot of functional enrichment analysis based on Gene Ontology (Biological Process) of co-expressed gene modules identified with SciGeneX in PBMC3k dataset. The y-axis represents the Gene Ontology terms, while the x-axis displays the gene ratio. Color represents the significance of their functional annotations.


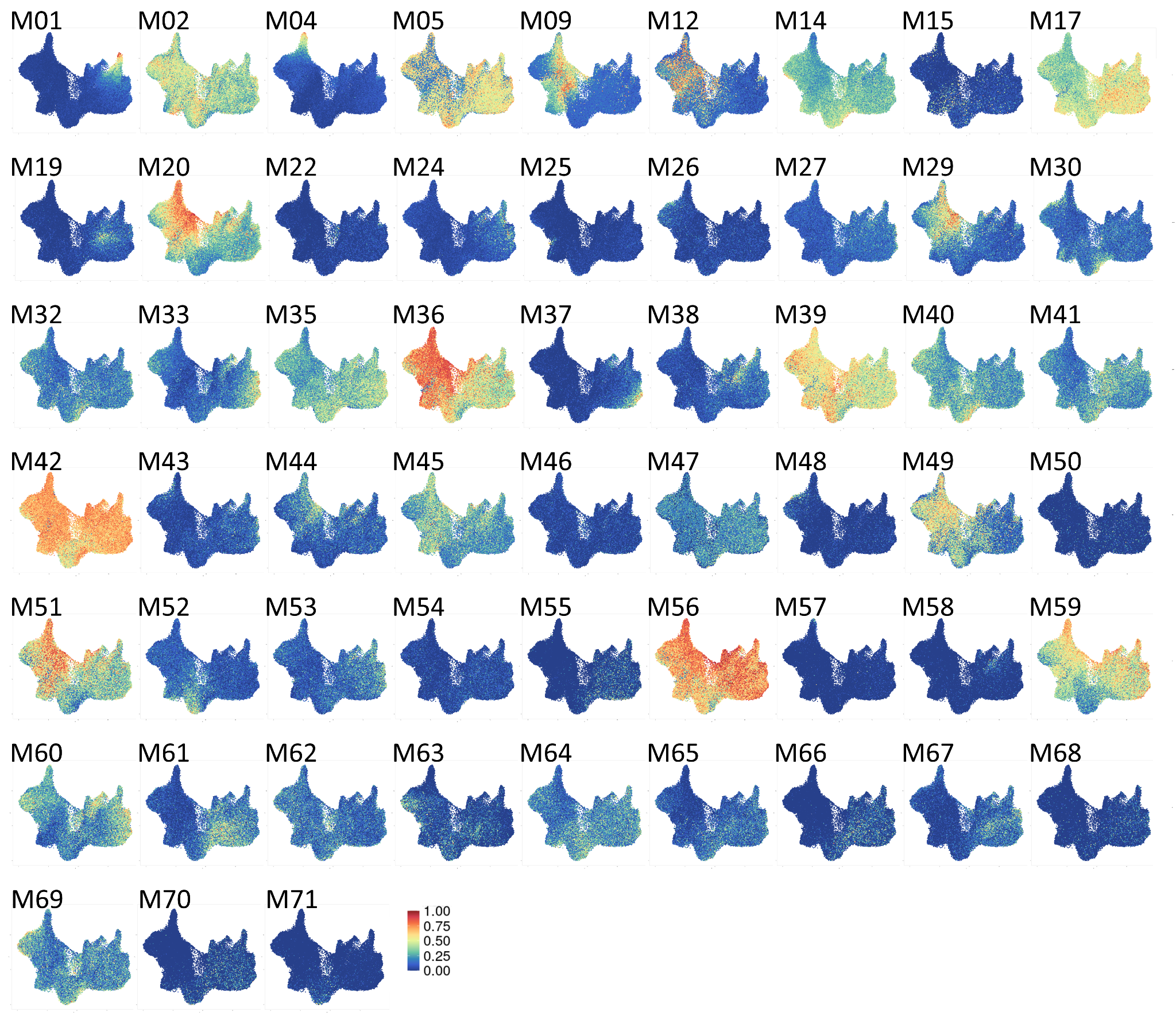


**Figure S7. UMAP visualization of AUCell scores for each gene module generated by SciGeneX in T cells trajectory dataset.** UMAP plots representing developing T cells from the T cell trajectory dataset. Cells are colored by the AUCell scores for each gene module generated by the SciGeneX algorithm.


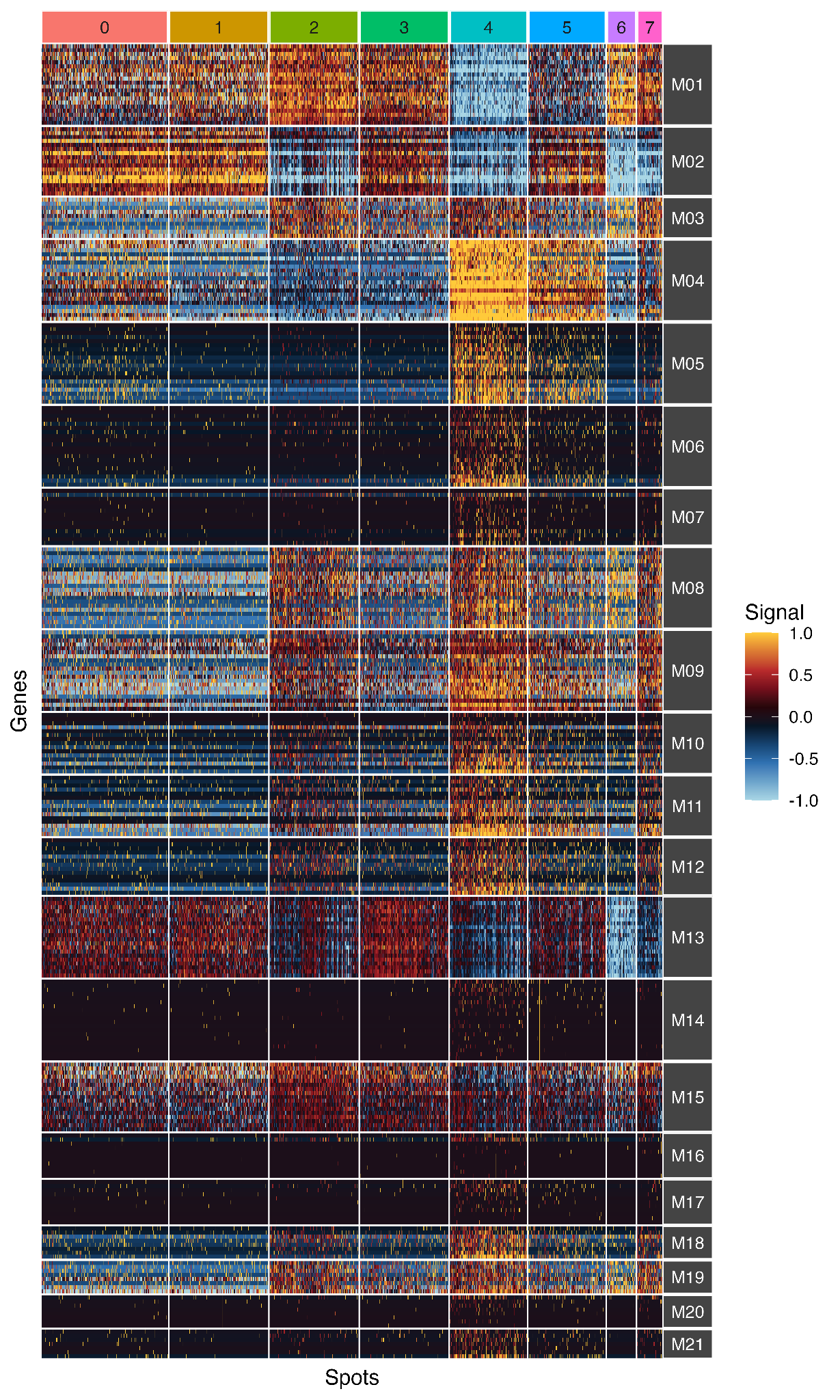


**Figure S8. Heatmap representation of co-expressed gene modules identified by SciGeneX in spatial transcriptomics of a human thymus section.** Heatmap displaying normalized expression levels of the top 20 genes within each co-expression module generated by the SciGeneX algorithm. The labels of the cell populations are shown on the top of the heatmap.


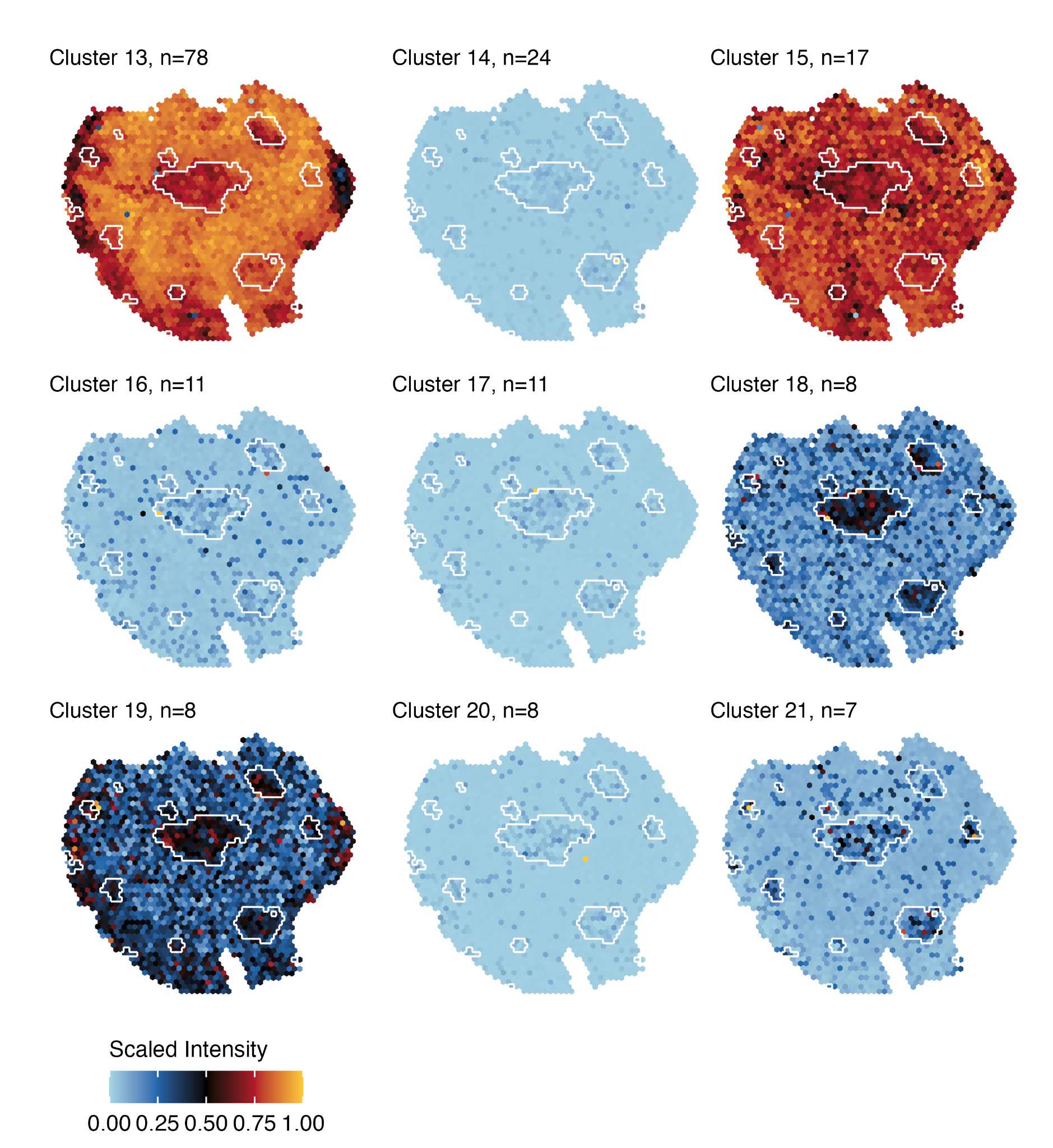


**Figure S9. Scaled expression intensity profiles of additional co-expressed gene modules on spatial transcriptomics dataset of a human thymus section.** Scaled expression intensity profiles of the co-expression modules not shown in Figure 7. Each spot on the spatial transcriptomics map represents the scaled intensity of co-expressed gene modules within the human thymus section.


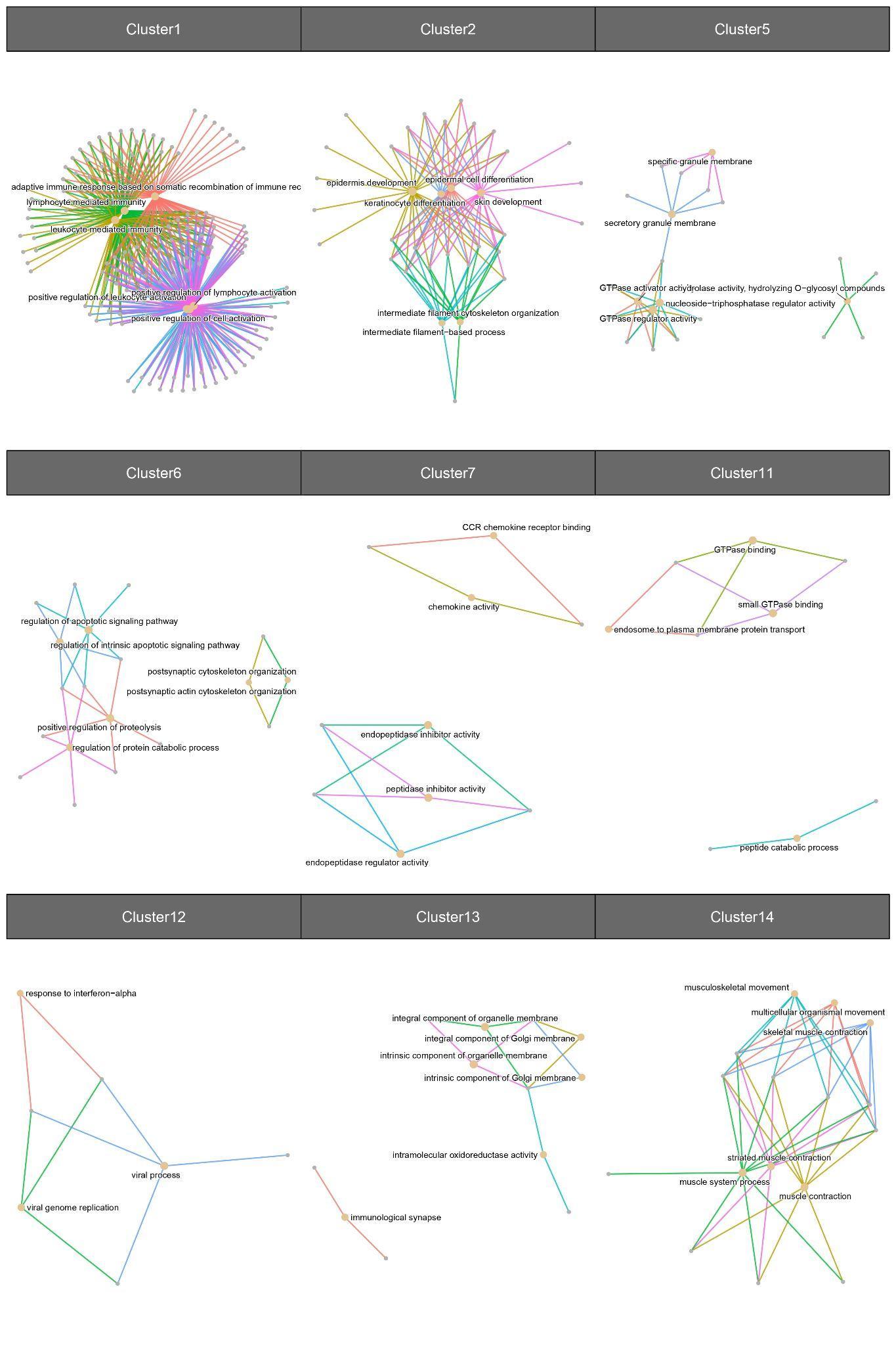


**Figure S10. Network representation of functional enrichment analysis of co-expressed gene modules obtained on spatial transcriptomics dataset of a human thymus section.** Network representation of the results obtained from the functional enrichment analysis based on Gene Ontology (Biological Process) of co-expressed gene modules identified within the spatial transcriptomics dataset of a human thymus section. The network visually represents the enriched biological processes associated with these gene modules.
